# Association between Dietary Intakes of Nitrate and Nitrite and the Risk of Hypertension and Chronic Kidney Disease: Tehran Lipid and Glucose Study

**DOI:** 10.3390/nu8120811

**Published:** 2016-12-21

**Authors:** Zahra Bahadoran, Parvin Mirmiran, Asghar Ghasemi, Mattias Carlström, Fereidoun Azizi, Farzad Hadaegh

**Affiliations:** 1 Nutrition and Endocrine Research Center, Research Institute for Endocrine Sciences, Shahid Beheshti University of Medical Sciences, Tehran 19395-4763, Iran; z.bahadoran@endocrine.ac.ir; 2 Endocrine Physiology Research Center, Research Institute for Endocrine Sciences, Shahid Beheshti University of Medical Sciences, Tehran 19395-4763, Iran; ghasemi@endocrine.ac.ir; 3 Department of Physiology and Pharmacology, Karolinska Institutet, Stockholm SE-171 76, Sweden; mattias.carlstrom@ki.se; 4 Endocrine Research Center, Research Institute for Endocrine Sciences, Shahid Beheshti University of Medical Sciences, Tehran 19395-4763, Iran; azizi@endocrine.ac.ir; 5 Prevention of Metabolic Disorders Research Center, Research Institute for Endocrine Sciences, Shahid Beheshti University of Medical Sciences, Tehran 19395-4763, Iran

**Keywords:** diet, nitrate, nitrite, hypertension, chronic kidney disease, glomerular filtration rate

## Abstract

Background and Aim: The association of habitual intakes of dietary nitrate (NO_3_^−^) and nitrite (NO_2_^−^) with blood pressure and renal function is not clear. Here, we investigated a potential effect of dietary NO_3_^−^ and NO_2_^−^ on the occurrence of hypertension (HTN) and chronic kidney disease (CKD). Methods: A total of 2799 Iranian adults aged ≥20 years, participating in the Tehran Lipid and Glucose Study (TLGS), were included and followed for a median of 5.8 years. Dietary intakes of NO_3_^−^ and NO_2_^−^ were estimated using a semi-quantitative food frequency questionnaire. Demographics, anthropometrics, blood pressure and biochemical variables were evaluated at baseline and during follow-up examinations. To identify the odds ratio (OR) and 95% confidence interval (CI) of HTN and CKD across tertile categories of residual energy-adjusted NO_3_^−^ and NO_2_^−^ intakes, multivariate logistic regression models were used. Results: Dietary intake of NO_3_^−^ had no significant association with the risk of HTN or CKD. Compared to the lowest tertile category (median intake < 6.04 mg/day), the highest intake (median intake ≥ 12.7 mg/day) of dietary NO_2_^−^ was accompanied with a significant reduced risk of HTN, in the fully adjusted model (OR = 0.58, 95% CI = 0.33–0.98; *p* for trend = 0.054). The highest compared to the lowest tertile of dietary NO_2_^−^ was also accompanied with a reduced risk of CKD (OR = 0.50, 95% CI = 0.24–0.89, *p* for trend = 0.07). Conclusion: Our findings indicated that higher intakes of NO_2_^−^ might be an independent dietary protective factor against the development of HTN and CKD, which are major risk factors for adverse cardiovascular events.

## 1. Introduction

Elevated blood pressure and renal dysfunction represent world-wide public health problems and are known to be major underlying causes for cardiovascular disease morbidity and mortality [1,2]. The key role of disrupted nitric oxide (NO) pathway, including either decreased production or reduced bioavailability of NO, is now well established in the pathogenesis of hypertension (HTN) and kidney disease [3,4,5]. The interaction of NO pathway with cardiorenal disease involves alterations of the renin-angiotensin (ANG) system, eicosanoid pathways, endothelines, cytokines, and regulators of inflammatory pathways [6]. NO deficiency has been associated with glomerular HTN and ischemia, glomerulosclerosis, proteinuria and kidney dysfunction [4,7,8].

In recent years, following the discovery of potential ability of inorganic nitrate (NO_3_^−^) and nitrite (NO_2_^−^) as an important back-up system for impaired NO synthase (NOS)-derived NO generation, the historical conception of the scientific community focused on the potential hazards of NO_3_^−^ and NO_2_^−^ exposures [9,10] shifted towards therapeutic properties of these compounds in cardiometabolic disorders [11,12,13,14,15,16,17,18,19,20,21]. Theoretically, reductions of NO_3_^−^ and NO_2_^−^ to NO could restore NO homeostasis, maintain the steady-state NO levels, and are considered as stable storage pools for NO-like bioactivity [22]. So, considering the role of NO as the key regulator of vascular homeostasis and natural vasodilator, supplementation with inorganic NO_3_^−^ and NO_2_^−^ have been investigated as potential therapeutic options in cardiovascular disease, including HTN, and in states renal dysfunction [23,24,25,26]. Currently, a large body of evidence supports a crucial role of NO_3_^−^ and NO_2_^−^ in the regulation and modulation of blood flow, endothelial function, and blood pressure [26,27,28]. Pre-clinical studies also confirm protective effects of NO_3_^−^ and NO_2_^−^ against ischemia-reperfusion injury, arterial stiffness, oxidative stress, inflammation and intimal thickness [26]. However, the nutritional aspects of the vasculo-protective effects of these anions are not clear and their long-term effects are still unknown; there is therefore a critical importance for good evidence to clarify the endpoints in the framework of epidemiological studies [29].

Following our findings regarding the protective effect of NO_3_^−^-containing vegetables against development of HTN, we speculated that the observed effect may be related to NO_3_^−^ [30]; after development of a valid database of NO_3_^−^/NO_2_^−^ content of food items [31], we expanded our hypothesis in the framework of the current study to clarify potential effects of NO_3_^−^/NO_2_^−^ on the risk of HTN and CKD.

To the best of our knowledge, the potential impact of dietary NO_3_^−^ and NO_2_^−^ on the occurrence of HTN and renal dysfunction has not yet been investigated in prospective longitudinal examination; such a setting could probably help to better justify the abovementioned experimental and clinical findings and provide more practical data for dietary recommendations regarding NO_3_^−^ and NO_2_^−^. The main focus in this study, therefore, was to ascertain whether regular intake of NO_3_^−^ and NO_2_^−^ could predict the occurrence of HTN and chronic kidney disease (CKD) among an Iranian population, during a 6-year follow-up.

## 2. Methods

### 2.1. Study Population

**Figure 1:**
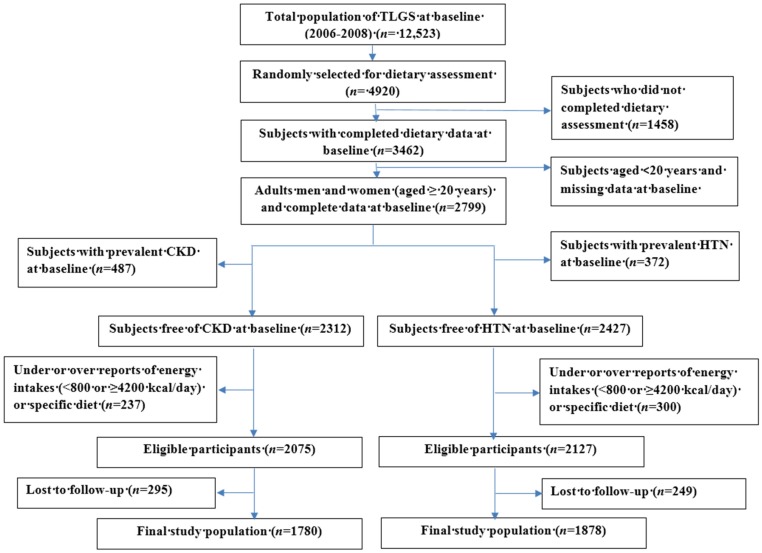
Flowchart of the study population, Tehran Lipid and Glucose Study (2006–2008 to 2012–2014).

This study was conducted within the framework of the Tehran Lipid and Glucose Study (TLGS), an ongoing community-based prospective study being conducted to investigate and prevent non-communicable diseases, in a representative sample in the district 13 of Tehran, the capital city of Iran [32]. During the third phase of the TLGS (2006–2008), a total of 12,523 subjects completed the examinations, of which 4920 were randomly selected for completing the dietary assessment based on their age and sex. The randomization was performed because of cost and complexity of dietary data collection in large populations and also the fact that this process is time consuming. Finally, the dietary data for 3462 subjects who agreed to participate and completed the food frequency questionnaire (FFQ) were available. The characteristics of participants who completed the validated FFQ were similar to those of the total population in the third phase of TLGS [33]. For the current analysis of, 2799 adult men and women (≥20 years) with complete data (demographics, anthropometrics, biochemicals and dietary data), were recruited. Two separate lines of exclusions were carried out for HTN and CKD as the outcomes. First, for the analysis of incident HTN, after exclusion of the participants with prevalent HTN at baseline (*n* = 372), and the participants with under- or over-reported energy intakes (<800 or ≥4200 kcal/day) or specific diet (including dietary recommendations for HTN, hyperlipidemia or diabetes) (*n* = 300), the remaining subjects were followed up to the fourth (2009–2011) and fifth TLGS (2012–2014) examinations. Participants who had no follow-up data (*n* = 249) were also excluded and final analyses was conducted on 1878 adults (806 men and 1072 women). Second, for the analysis of incident CKD, after exclusions included individuals with prevalent CKD at baseline (*n* = 487), unusual diet (*n* = 237), along with 295 who did not attend any follow-up examinations, resulting in a total number of 1780 adults (727 men and 1053 women). A flowchart of the study population is shown in Figure 1. Written informed consents were obtained from all participants, and the study protocol was approved by the ethics research council of the Research Institute for Endocrine Sciences, Shahid Beheshti University of Medical Sciences in Teheran (18ECRIES93/11/26).

### 2.2. Demographic, Anthropometric and Clinical Measures

Trained interviewers collected information using standard questionnaires. Detailed measurements of variables in TLGS have been reported elsewhere [32]. Smoking status was obtained using face-to-face interviews; subjects who smoked daily or occasionally were considered current smokers. Weight was measured to the nearest 100 g using digital scales, while the subjects were minimally clothed, without shoes. Height was measured to the nearest 0.5 cm, in a standing position without shoes, using a tape meter. Body mass index (BMI) was calculated as weight (kg) divided by square of the height (m^2^). Waist circumference (WC) was measured to the nearest 0.1 cm, midway between the lower border of the ribs and the iliac crest at the widest portion, over light clothing, using a soft measuring tape, without any pressure to the body.

For measurements of systolic (SBP) and diastolic blood pressure (DBP), after a 15-min rest in upright position, two measurements of blood pressure were taken on the right arm, during a standardized mercury sphygmomanometer; the mean of the two measurements was considered as the participant’s blood pressure.

### 2.3. Biochemical Measures

Fasting blood samples were taken after 12–14 h, from all study participants at baseline and at follow-up phases. Serum creatinine levels were assayed using kinetic colorimetric Jaffe method. Fasting plasma glucose (FPG) was measured by the enzymatic colorimetric method using glucose oxidase. The standard 2-h post-challenge plasma glucose (2 h-PCPG) test was performed using oral administration of 82.5 g glucose monohydrate solution (equivalent to 75 g anhydrous glucose) for all individuals who were not on glucose lowering drugs.

Triglyceride (TG) level was measured by enzymatic colorimetric analysis with glycerol phosphate oxidase. High-density lipoprotein cholesterol (HDL-C) was measured after precipitation of the apolipoprotein B containing lipoproteins with phosphotungstic acid. Analyses were performed using Pars Azmoon kits (Pars Azmoon Inc., Tehran, Iran) and a Selectra 2 auto-analyzer (Vital Scientific, Spankeren, The Netherlands). Both inter- and intra-assay coefficients of variation of all assays were <5%.

To develop a validation study for dietary NO_3_^−^ and NO_2_^−^, urine NO_3_^−^ and NO_2_^−^ concentration was measured in a sub-sample of population (*n* = 251), by a rapid, simple spectrophotometric method [34,35,36].

### 2.4. Dietary Assessment

A validated 168-item food frequency questionnaire (FFQ) was used to assess typical food intakes over the previous year. Trained dietitians, with at least 5 years of experience in the TLGS survey, asked participants to designate their intake frequency for each food item consumed during the past year on a daily, weekly, or monthly basis. Portion sizes of consumed foods reported in household measures were then converted to grams [33]. The validity of the food frequency questionnaire has been previously evaluated by comparing food groups and nutrient values determined from the questionnaire with values estimated from the average of twelve 24-h dietary recall surveys and the reliability has been assessed by comparing energy and nutrient intakes from two FFQ; Pearson correlation coefficients and intra-class correlation for energy and nutrients showed acceptable agreements between FFQ and twelve 24-h dietary recall surveys, and FFQ1 and FFQ2 [37].

Since the Iranian Food Composition Table is incomplete, and has limited data on nutrient content of raw foods and beverages, to analyze foods and beverages for their energy and nutrient content (except NO_3_^−^ andNO_2_^−^), the US Department of Agriculture Food Composition Table was used [38].

Food composition values for NO_3_^−^ andNO_2_^−^ were derived from a recent survey conducted on frequently consumed food items among Iranians. Briefly, we determined the NO_3_^−^ andNO_2_^−^ contents of 87 food items including grains, legumes, fruits and vegetables, dairy products, meats and processed meats using validated spectrophotometric methods [39]. A relatively high NO_3_^−^ concentration was observed in breads (~50.0 mg·100 g^−1^). Mean ranges of NO_3_^−^ and NO_2_^−^ in fruits were 7.50–46.8 and 0.15–0.71 mg·100 g^−1^, respectively. Vegetables with the highest NO_3_^−^ concentrations included radish (626 mg·100 g^−1^), beetroot (495 mg·100 g^−1^), tarragon (424 mg·100 g^−1^), lettuce (365 mg·100 g^−1^), mint (279 mg·100 g^−1^), and celery (261 mg·100 g^−1^). The levels of NO_2_^−^ in vegetables ranged 0.21–0.74 mg·100 g^−1^. In dairy products, mean NO_3_^−^ and NO_2_^−^ content ranged 0.14–0.45 and 1.26–5.75 mg·100 g^−1^. Mean NO_3_^−^ and NO_2_^−^ concentrations in meats and processed meats were 5.56–19.4 and 2.93–13.9 mg·100 g^−1^, respectively.

### 2.5. Validity of NO_3_^−^ and NO_2_^−^ Estimation by FFQ

Among a subsample (*n* = 251) of participants in the TLGS population, after adjustment for intra- to inter-individual variance and other potential confounders including age, BMI and serum Cr levels, a good agreement was observed between dietary intakes of NO_3_^−^ and NO_2_^−^ and their urinary values (*r* = 0.59, 95% CI = 0.49, 0.67, and *r* = 0.29, 95% CI = 0.17, 0.41).

### 2.6. Definition of Terms and Outcomes

The HTN was defined as SBP ≥ 140 or mmHg DBP ≥ 90 mmHg, or self-reported taking blood pressure lowering medication [40].

Incident CKD was defined as estimated glomerular filtration rate (eGFR) <60 mL/min/1.73 m^2^ occurring at any time during the follow-up period; this corresponds to stage 3 to stage 5 CKD based on the Kidney Disease Outcomes and Quality Initiative guidelines [41]. To calculate eGFR, the CKD Epidemiology Collaboration (EPI) equation was used. As a single equation CKD-EPI has been expressed as follows:

eGFR = 141 × min(S_cr_/κ, 1)^α^ × max(S_cr_/κ, 1)^−1.209^ × 0.993^age^ × 1.018 (if female) × 1.159 (if black) (1)

In this equation, S_cr_ is serum Cr in mg/dL; κ is 0.7 and 0.9 for men and women, respectively, α is −0.329 and −0.411 for men and women, respectively; min indicates the minimum of S_cr_/κ or 1, and max indicates maximum of S_cr_/κ or 1 [42].

The family history of premature cardiovascular disease was obtained by asking participants whether any member in their first-degree relatives had experienced a fatal or non-fatal myocardial infarction, stroke, or sudden cardiac arrest; the event was considered premature if it occurred in persons <55 years of age in male relatives and <65 years of age in female relative [43]. Type 2 diabetes (T2D) was defined as FPG ≥ 7 mmol/L or 2 h-PCPG ≥ 11.1 mmol/L, or taking antidiabetic medication [44].

### 2.7. Statistical Analyses

Dietary intakes of NO_3_^−^ and NO_2_^−^ and other nutrients were adjusted for total energy intake, according to residuals methods [45]. The incidence of HTN and CKD over the follow-up period was considered as a dichotomous variable (yes/no) in the models. The mean and standard deviation (SD) values, and the frequency (%) of baseline characteristics of the participants with and without HTN and CKD were compared using independent *t* test or chi square test, respectively. A univariate analysis was performed to identify potential covariates and the variables with P_E_ < 0.2 in the univariate analyses were selected for the final multivariable models. Potential confounding variables adjusted in the final regression model were included baseline SBP (mmHg), baseline DBP (mmHg), WC (cm), family history of premature cardiovascular disease (yes/no), smoking (yes/no), lipid-lowering drugs (yes/no), aspirin (yes/no), dietary intakes of fiber (g/day), fat (g/day), potassium (mg/day) and sodium (mg/day) for HTN [43], and age (years), sex (male/female), type 2 diabetes (yes/no), HTN (yes/no), eGFR (mL/min/1.73 m^2^), smoking (yes/no), dietary intakes of fat (g/day), protein (g/day), potassium (mg/day) and sodium (mg/day) for CKD [46]. The association between different intake levels of NO_3_^−^ and NO_2_^−^ intake with incident HTN and CKD was assessed by multivariate adjusted odds ratios (ORs) with 95% confidence interval (CI) using binary logistic regression analysis. For risk covariates with more than 2 categories, the first category was considered as the reference group, in the model. To assess the overall trends of odds ratios, the median of each tertile was used as a continuous variable in logistic regression models. All statistical analyses were conducted using SPSS (Version 16.0, IBM; Chicago, IL, USA), and *p* values < 0.05 were considered significant.

## 3. Results

Mean (SD) intakes of dietary NO_3_^−^ and NO_2_^−^ was 455 (188) and 9.4 (3.6) mg/day, respectively. In our population, the major contributors to NO_3_^−^ intakes were vegetables (46.1%) and grains (28.8%). Dietary intakes of NO_2_^−^ from animal sources accounted for 42.4% of daily mean intake of NO_2_^−^ and the remainder of NO_2_^−^ intake was derived from plant sources. The major contributors to NO_2_^−^ intake were white rice (17.1%), chicken meat (11.7%), yogurt (6.6%), tomato (5.3%), sausages (4.7%), lamb meat (3.5%), cucumber (3.3%).

Baseline characteristics and dietary intakes of the participants are compared across tertile categories of dietary intakes of NO_3_^−^ in HTN-free subjects, in Table 1 and Table 2, respectively. Participants in the highest compared to the lowest tertile of NO_3_^−^, were less likely to be smoker (8.9 vs. 13.1, *p* < 0.05); there was no significant difference in lipid lowering drug and aspirin intakes, anthropometric measures, systolic and diastolic blood pressures, FPG and TG to HDL-C ratio across NO_3_^−^ tertiles. All components of the diet had increasing trend across increasing intakes of NO_3_^−^. There was no significant difference in the rate of incident-case of HTN across NO_3_^−^ and NO_2_^−^ tertiles, after 5.8 years of follow-up. Baseline characteristics of the participants are compared across tertile categories of dietary intakes of NO_3_^−^ and NO_2_^−^ in CKD-free subjects, in Supplementary Materials Table S1. Participants in the highest compared to the lowest tertile of NO_3_^−^, were more likely to be older (36.7 vs. 30.6 years, *p* < 0.05), and had lower serum creatinine levels (90.8 vs. 93.2 µmol/L, *p* < 0.05); there was a non-significant lower rate of incident-CKD in the highest compared to the lowest tertile of dietary NO_3_^−^ (16.7% vs. 19.6%) and NO_2_^−^ (17.0% vs. 18.5%) intakes.

Baseline characteristics of the study participants for incident HTN according to outcome status are shown in Supplementary Materials Table S2. Mean (SD) age of the study participants was 36.6 (12.4) years and 42.9% were men. Mean (SD) BMI was 26.3 (4.7) kg/m^2^, at baseline. Overall, 291 new cases HTN were identified after a median follow-up of 5.8 years; the corresponding cumulative incidence was 15.5%. Compare with non-HTNs, hypertensive subjects were more likely to be older, and had higher BMI, WC, blood pressures, FPG, TG to HDL-C ratio, prevalent T2D, creatinine levels and lower eGFR (*p* for all < 0.05). Mean (SD) baseline intake of NO_3_^−^ and NO_2_^−^ was 455 ± 188 and 9.47 ± 3.61 mg/day, and there was no difference in dietary intakes of NO_3_^−^ andNO_2_^−^ between the groups.

Baseline characteristics of the participants for incident CKD are shown in Supplementary Materials Table S3. Mean (SD) age of the study participants was 33.9 (15.4) years and 40.8% were men. Mean (SD) BMI was 27.4 (4.8) kg/m^2^, at baseline. Over a median 5.8 years of follow-up, a total of 306 cases of CKD were diagnosed (cumulative incidence rate = 17.2%). The CKD patients had higher BMI, WC, blood pressures, FPG, TG to HDL-C ratio, and lower eGFR rate, at baseline (*p* for all < 0.05). Compared to CKD patients, non-CKD subjects had higher intake of NO_3_^−^ (467 vs. 443 mg/day, *p* = 0.02) at baseline, whereas no significant difference was observed in NO_2_^−^ intake between the groups.

Association between NO_3_^−^ and NO_2_^−^ intake and the risk of HTN after 5.8 years of follow-up are shown in Table 3. We did not observe any significant association between intake of NO_3_^−^ and the risk of HTN in the logistic regression models. Compared to the lowest tertile category (median intake < 6.04 mg/day), the highest intake (median intake ≥ 12.7 mg/day) of dietary NO_2_^−^ was accompanied with a significant reduced risk of HTN, in the fully adjusted model (OR = 0.58, 95% CI = 0.33–0.98; *p* for trend = 0.054).

The incidence of CKD across tertile categories of NO_3_^−^ and NO_2_^−^ intake are shown in Table 4. After adjustment of major potential confounding variables, dietary intake of NO_3_^−^ had no significant association with the risk of CKD whereas highest compared to the lowest tertile of dietary NO_2_^−^ was accompanied with a reduced risk of CKD (OR = 0.50, 95% CI = 0.24–0.89, *p* for trend = 0.07).

## 4. Discussion

In this longitudinal study, we investigated the potential impact of habitual dietary NO_3_^−^ and NO_2_^−^ intake on the risk of HTN and CKD, in the framework of a population-based study, for the first time. Higher dietary NO_2_^−^ intake was significantly associated with a reduced risk of HTN and CKD, independent of the major potential risk factors. Compared to CKD patients, non-CKD subjects had higher intake of NO_3_^−^ (467 vs. 443 mg/day, *p* = 0.02) at baseline; however, dietary NO_3_^−^ intake was not related to incidence of either HTN of CKD after a median 5.8 years of follow-up.

Most recent findings imply beneficial cardio-renal protective and antihypertensive outcomes following short-term administration of inorganic NO_3_^−^ [16,19,24,47]. The underlying mechanisms for the favorable effects of inorganic NO_3_^−^ and NO_2_^−^ in human subjects are still not fully understood, but it has been proposed that NO_2_^−^ could be a stable endocrine carrier and transducer of NO-like bioactivity within the circulation; systemic vasodilatation through the NO-cGMP pathway has been suggested as the acute effects of dietary NO_3_^−^ and NO_2_^−^ [28,29]. The novel mechanisms recently investigated in a model of natural aging-related cardiovascular and metabolic abnormalities, suggest that inorganic NO_3_^−^ mediates its therapeutic effects through restored cGMP signaling and increased NO bioavailability, decreased ANG II type 1 receptor expression, improved endothelial function, increased insulin release and reduced NADPH oxidase activity and superoxide generation [25]. Beneficial effects of NO_3_^−^ and NO_2_^−^ on renal function may be explained by promoting the NO_3_^−^–NO_2_^−^–NO pathway, attenuation of ANG II-induced hypertension, and reducing constriction of renal afferent arterioles [48,49]. It also has been shown that NO_3_^−^ supplementation could normalize elevated plasma creatinine levels and improve glomerular function during aging [25] and prevent renal dysfunction in experimental models of compromised kidney function and cardiovascular disease [24]. In addition, experimental studies have indicated that inorganic nitrite may protect from kidney injuries following acute ischemia-reperfusion [50,51]. Hence, the above mentioned mechanisms might justify 42% and 50% decreased risk of HTN and CKD in relation to dietary intakes of NO_2_^−^ more than ~10 mg/day, in our study population.

In contrast to the vast majority of experimental findings indicating renoprotective properties of NO_3_^−^, there have been some concerns regarding its harmful effects for humans, especially when used in high doses to improve exercise performance. To address this challenging issue, a recent clinical study investigated the effects of potassium NO_3_^−^ (450 mg/day) on GFR, and urine output for creatinine, albumin, and urea, in young male during a cycling exercise condition. This study reported no adverse effects on renal function, over one week period of NO_3_^−^ supplementation [52]. Currently, there are no further clinical studies to confirm or reject beneficial effects of inorganic NO_3_^−^ and NO_2_^−^ on kidney function.

An overview of the current literature displays lack of epidemiological evidence regarding cardiorenal outcomes of NO_3_^−^ and NO_2_^−^ in the context of daily dietary intake. The only relevant studies in this case, was our previous cohort with a 3-year follow-up that showed a protective effect against HTN and no significant impact on CKD following higher consumption of NO_3_^−^-containing vegetables [30,53]. Lack of information regarding true NO_3_^−^/NO_2_^−^ content of the vegetables was an important limitation of these works; we also did not observe any difference between categories of NO_3_^−^-containing vegetables (including low-, medium- and high-NO_3_^−^) in relation to HTN, so we concluded that other bioactive compounds, including phytochemicals and antioxidant components, may be involved in the hypotensive effect of these vegetables. In the current study, NO_3_^−^ from vegetable sources was not related to risk of HTN (OR = 0.97, 95% CI = 0.67–1.42, and OR = 0.98, 95% CI = 0.63–1.52, in the second and third quartile categories, respectively) and CKD (OR = 1.31, 95% CI = 0.88–1.93, and OR = 0.93, 95% CI = 0.57–1.50, in the second and third quartile categories, respectively).

The usual dietary consumption of NO_3_^−^ and NO_2_^−^ in our study was higher than in other previous reports such as the Shanghai Women’s Health Study, National Institutes of Health/American Association of Retired Persons (NIH-AAPR) diet and health study, which estimated dietary intakes of ~300 and 100 mg/day for NO_3_^−^ and 1.4 and 1.0 mg/day for NO_2_^−^ intake [54,55]. Moreover, our intakes was approximately twice the acceptable daily intake (ADI) values, defined as 3.7 and 0.06 mg/kg body weight for NO_3_^−^ and NO_2_^−^, respectively [56]. Major sources of NO_3_^−^ intakes were grains and vegetables; due to a relatively high NO_3_^−^ concentration in our traditional and industrial breads (50.0 mg·100 g^−1^) [31], and high proportion of breads (320 g/day) in the dietary pattern of the Iranian population [57], NO_3_^−^ exposure from this food group was considerable in our population. In our previous study we indicated that mean NO_3_^−^ levels in 68.3% of lettuce, 92.5% of potato, 90.9% of radish, and 51.0% of cabbage samples exceeded the maximum limits legislated by European countries for trade of vegetables; moreover, mean NO_2_^−^ contents of fruit samples were also relatively high [31]. High intake of NO_3_^−^ and NO_2_^−^ in our population, therefore, may be attributed to either high content of NO_3_^−^/NO_2_^−^ in Iranian foods or high intake of NO_3_^−^/NO_2_^−^-containing foods. Hence, considering the fact that most dietary substances are rather low in NO_2_^−^, and that the vast majority of NO_2_^−^ is likely to be derived from reduction of dietary NO_3_^−^ [28], rather than dietary NO_2_^−^ per se, the correlation between dietary NO_2_^−^ and its urinary values, we observed in a validity study, was rather week.

Our study had some strengths and limitations. The large, prospective population-based design, a high rate of follow-up completeness, and use of a validated comprehensive FFQ to assess regular dietary intakes of the participants provided us an opportunity to examine the potential effect of NO_3_^−^ and NO_2_^−^ on the risk of HTN and CKD, relationship that have not been previously reported. Estimation of NO_3_^−^/NO_2_^−^ based on measured values in frequently consumed food items among our population [31], compared to other previous cohorts which have relied on historic literature values, may fully reflect the accurate NO_3_^−^/NO_2_^−^ exposure from the diet.

We could not estimate NO_3_^−^/NO_2_^−^ exposure from drinking water due to lack of data for drinking water NO_3_^−^/NO_2_^−^ contents and individuals’ information regarding water intake, at baseline. However, previous studies showed that NO_3_^−^/NO_2_^−^ concentration of drinking water was lower than the standard limits (50 mg/L) [58]; considering the low amount of water intake among the Iranian population (~0.96 L) [59], it seems that NO_3_^−^/NO_2_^−^ intakes from drinking water are relatively low. Furthermore, our recent study in district 13 of Tehran showed that NO_3_^−^ and NO_2_^−^ levels of drinking water was 32.8 ± 9.9 and 2.6 ± 0.5 mg/L, respectively, and estimation of NO_3_^−^/NO_2_^−^ intakes from drinking water in a subsample of TLGS population showed a relatively low contribution of drinking water in overall NO_3_^−^/NO_2_^−^ exposure compared to its dietary sources (6.7% and 26.6% for NO_3_^−^ and NO_2_^−^, respectively).

Estimates of NO_2_^−^ from meat are likely to be inaccurate as most of the NO_2_^−^ forms nitrosylmyoglobin. Furthermore, due to potential changes in an individual’s diet and NO_3_^−^/NO_2_^−^ content of food items, as well as changes in other risk factors of HTN and CKD over the time of study follow-up, some degree of misclassification might have occurred which could lead to biased estimated hazard ratios towards the null, as inherent to any prospective study.

In conclusion, this prospective study suggests that a higher intake of dietary NO_2_^−^ can decrease the risk of developing both HTN and CKD. Although higher intake of NO_3_^−^ was associated with lower incidence of CKD at baseline, we did not find that differences in NO_3_^−^ intake influenced incidence of HTN or CKD during the study period of 6 years. Our findings, especially in the case of NO_2_^−^ supplementation, support previous experimental and clinical studies that suggest the therapeutic value of boosting the NO_3_^−^–NO_2_^−^–NO pathway.

## 5. Perspectives

In this study, dietary NO_2_^−^ intake had a protective effect against CKD and HTN, and both of them are associated with decreased nitric oxide availability. Dietary NO_3_^−^ and NO_2_^−^ could act as precursors for nitric oxide production in case of its deficiency. It seems that intake of NO_3_^−^/NO_2_^−^ should be taken into consideration in dietary assessments, in particular in patients with CKD and HTN. In addition, since NO_3_^−^/NO_2_^−^ therapy could easily be achieved through nutrition-based interventions, it could be speculated that such intervention contributes to the future management of HTN and kidney diseases.

## Figures and Tables

**Table 1:** Characteristics across tertile categories of dietary nitrate and nitrite intakes in hypertension (HTN)-free subjects at baseline (*n* = 1878).

	Dietary Nitrate (mg/Day)			Dietary Nitrite (mg/Day)		
	Tertile 1	Tertile 2	Tertile 3	Tertile 1	Tertile 2	Tertile 3
	<359	359–505	≥505	<7.5	7.5–10.6	≥10.6
Age (years)	36.3 ± 12.1	37.3 ± 12.2	37.7 ± 12.8	37.8 ± 12.5	37.5 ± 12.2	35.8 ± 12.3
Men (%)	39.9	45.7	43.1	37.5	41.7	49.5 *
Smoking (%)	13.1	13.9	8.9 *	10.6	13.1	12.4
Lipid lowering drugs (%)	1.1	2.7	2.5	1.6	2.9	2.2
Aspirin (%)	3.7	5.9	5.4	5.3	4.3	5.4
Body mass index (kg/m^2^)	26.0 ± 4.6	26.3 ± 4.5	26.5 ± 4.8	26.1 ± 4.8	26.3 ± 4.6	26.4 ± 4.7
Waist circumference (cm)	86.1 ± 13.3	78.5 ± 12.4	88.1 ± 13.1	86.2 ± 13.2	87.4 ± 12.2	88.1 ± 13.4
SBP (mmHg)	106 ± 12.0	107 ± 11.3	107 ± 11.9	106 ± 11.3	107 ± 12.5	107 ± 11.5
DBP (mmHg)	70.2 ± 9.0	71.2 ± 8.6	71.1 ± 8.8	70.3 ± 8.8	71.1 ± 8.7	71.0 ± 8.9
FPG (mmol/L)	86.9 ± 13.1	88.1 ± 14.6	88.0 ± 16.7	88.3 ± 17.8	87.7 ± 13.8	86.2 ± 12.8
TG to HDL-C ratio	3.2 ± 2.6	3.4 ± 2.6	3.3 ± 2.5	3.2 ± 2.4	3.3 ± 2.4	3.4 ± 2.8
Serum creatinine (μmol/L)	88.6 ± 12.6	89.4 ± 12.2	90.0 ± 14.4	88.1 ± 14.0	89.1 ± 13.0	90.0 ± 12.0
eGFR (mL/min/1.73 m^2^)	81.2 ± 13.0	81.0 ± 13.0	80.0 ± 13.7	80.3 ± 13.2	80.6 ± 13.7	81.6 ± 12.7
Dietary NO_3_^−^ (mg/day)	276 ± 58.7	428 ± 41.9	660 ± 162 *	314 ± 106	447 ± 117	604 ± 196 *
Dietary NO_2_^−^ (mg/day)	6.7 ± 2.4	9.2 ± 2.2	12.4 ± 3.5 *	5.9 ± 1.1	9.0 ± 0.8	13.5 ± 2.8 *
Incident case of HTN after 5.8 years (%)	16.7	18.6	17.0	17.1	14.7	14.7

Data are mean ± SD (unless stated otherwise); Analysis of variance or chi-square test were used for continuous and categorical variables, respectively; * *p* < 0.05; SBP: Systolic blood pressure; DBP: Diastolic blood pressure; FPG: Fasting plasma glucose; TG: Triglycerides; eGFR: Estimated glomerular filtration rate; HTN: Hypertension.

**Table 2:** Dietary intakes of across tertile categories of dietary nitrate intakes in HTN-free subjects at baseline (*n* = 1878).

	Tertile 1	Tertile 2	Tertile 3
	<359 mg/Day	359–505 mg/Day	≥505 mg/Day
Energy (kcal/day)	1708 ± 473	2273 ± 536	2809 ± 640 *
Carbohydrate (g/day)	234 ± 63.9	322 ± 77.9	414 ± 108 *
Total fats (g/day)	64.1 ± 26.7	81.3 ± 28.2	93.8 ± 31.6 *
Proteins (g/day)	57.7 ± 18.9	75.9 ± 19.8	97.1 ± 27.1 *
Total fiber (g/day)	24.1 ± 12.5	36.5 ± 15.7	49.0 ± 21.2 *
Sodium (g/day)	4.1 ± 0.6	4.5 ± 1.0	5.4 ± 1.2 *
Potassium (g/day)	2.5 ± 0.7	3.6 ± 1.4	5.1 ± 1.8 *
Vegetables (g/day)	90.2 ± 42.1	155 ± 57.9	269 ± 142 *
Fruits (g/day)	184 ± 130	319 ± 196	501 ± 320 *
Dairy (g/day)	343 ± 248	450 ± 261	525 ± 331 *
Legumes (g/day)	10.9 ± 12.5	15.7 ± 19.8	20.7 ± 27.4 *
Grains (g/day)	297 ± 127	402 ± 174	491 ± 282 *
Meats (g/day)	42.7 ± 35.2	52.0 ± 32.4	67.1 ± 60.4 *
Processed meats (g/day)	9.5 ± 11.2	11.2 ± 10.9	13.8 ± 17.1 *

Data are mean ± SD; Analysis of variance was used (* *p* < 0.05).

**Table 3:** The association of dietary nitrate and nitrite intakes and the risk of HTN after 6-years of follow-up: Tehran Lipid and Glucose Study 2006–2008 to 2012–2014.

	Tertile 2	Tertile 3
Dietary NO_3_^−^ (mg/day)	359–505	≥505
Crude	1.13 (0.83–1.53)	1.02 (0.75–1.39)
Model 1	1.06 (0.73–1.43)	0.81 (0.58–1.17)
Model 2	1.02 (0.68–1.51)	0.81 (0.48–1.38)
Dietary NO_2_^−^ (mg/day)	7.58–10.6	≥10.6
Crude	0.85 (0.61–1.18)	0.86 (0.56–1.33)
Model 1	0.66 (0.45–1.00)	0.58 (0.34–0.99)
Model 2	0.66 (0.44–1.00)	0.58 (0.33–0.98)

Odds ratio (95% CI); logistic regression models were used. The first tertile of NO_3_^−^ (<359 mg/day) and NO_2_^−^ intake (<7.58 mg/day) was considered as reference group. Model 1: Adjusted for age (years), sex (male/female), systolic and diastolic blood pressure (mmHg), waist circumference (cm), family history of premature cardiovascular disease (yes/no), and smoking (yes/no), lipid-lowering drugs (yes/no), aspirin (yes/no); Model 2: Additional adjustment for dietary intake of total fiber (g/day), fat (g/day), potassium (mg/day), and sodium (mg/day). Median intake of dietary NO_3_^−^ was 288, 428, and 613 mg/day, in the first, second, and third tertile categories. Median intake of dietary NO_2_^−^ was 6.04, 9.00, and 12.7 mg/day, in the first, second, and third tertile categories. HTN: Hypertension.

**Table 4:** The association of dietary nitrate and nitrite intakes and the risk of chronic kidney disease (CKD) after 6-years of follow-up: Tehran Lipid and Glucose Study 2006–2008 to 2012–2014.

	Tertile 2	Tertile 3
Dietary NO_3_^−^ (mg/day)	365–511	≥511
Crude	0.83 (0.61–1.14)	0.76 (0.52–1.12)
Model 1	1.07 (0.71–1.60)	0.78 (0.48–1.28)
Model 2	1.04 (0.68–1.57)	0.76 (0.43–1.24)
Dietary NO_2_^−^ (mg/day)	7.69–10.7	≥10.7
Crude	0.95 (0.69–1.31)	0.87 (0.57–1.33)
Model 1	0.79 (0.52–1.18)	0.55 (0.32–0.93)
Model 2	0.76 (0.50–1.13)	0.50 (0.24–0.89)

Odds ratio (95% CI); logistic regression models were used; the first tertile of NO_3_^−^ (<365 mg/day) and NO_2_^−^ intake (<7.69 mg/day) was considered as reference group; the number of case/total was 116/593, 103/594, and 99/593 in the first, second, and third tertile categories of dietary nitrate intakes. The number of case/total was 110/593, 107/594, and 101/593 in the first, second, and third tertile categories of dietary nitrite intakes; Model 1: Adjusted for age (years), sex (male/female), diabetes (yes/no), hypertension (yes/no), eGFR (mL/min/1.73 m^2^), and smoking (yes/no); Model 2: Additional adjustment for dietary intake protein (g/day), fat (g/day), potassium (mg/day), and sodium (mg/day); Median intake of dietary NO_3_^−^ was 291, 431, and 619 mg/day, in the first, second, and third tertile categories; Median intake of dietary NO_2_^−^ was 6.14, 9.08, and 12.8 mg/day, in the first, second, and third tertile categories.

## Supplementary Materials

The following are available online at http://www.mdpi.com/2072-6643/8/12/811/s1, Table S1. Characteristics across tertile categories of dietary nitrate and nitrite intakes in CKD-free subjects at baseline (*n* = 1780), Table S2. Baseline characteristics of the participants according to follow-up HTN status: Tehran Lipid and Glucose Study 2006–2008 to 2012–2014, Table S3. Baseline characteristics of the participants according to follow-up CKD status: Tehran Lipid and Glucose Study 2006–2008 to 2012–2014.
