# Supplementary material for: Association between Dietary Intakes of Nitrate and Nitrite and the Risk of Hypertension and Chronic Kidney Disease: Tehran Lipid and Glucose Study
Source: Nutrients. 2016 Dec 21;8(12):811. doi: 10.3390/nu8120811 (PMC5188466; doi:10.3390/nu8120811)
Supplement: Supplementary file 1 [file nutrients-08-00811-s001.docx]

Supplementary Materials: Association between Dietary Intakes of Nitrate and Nitrite and the Risk of Hypertension and Chronic Kidney Disease: Tehran Lipid and Glucose Study

Zahra Bahadoran, Parvin Mirmiran, Asghar Ghasemi, Mattias Carlström, Fereidoun Azizi and Farzad Hadaegh

**Table S1.** Characteristics across tertile categories of dietary nitrate and nitrite intakes in CKD-free subjects at baseline (*n* = 1780).

|  | **Dietary Nitrate (mg/Day)** | | | **Dietary Nitrite (mg/Day)** | | |
| --- | --- | --- | --- | --- | --- | --- |
|  | **Tertile 1  <365** | **Tertile 2  365–510** | **Tertile 3  ≥510** | **Tertile 1  <7.6** | **Tertile 2  7.6–10.7** | **Tertile 3  ≥10.7** |
| Age (year) | 30.6 ± 14.9 | 34.6 ± 15.1 | 36.7 ± 15.5 * | 32.6 ± 15.6 | 35.2 ± 15.7 | 33.9 ± 14.7 |
| Men (%) | 42.7 | 41.1 | 38.8 | 45.0 | 41.6 | 35.9 * |
| Smoking (%) | 8.7 | 10.2 | 7.5 | 9.9 | 10.4 | 6.2 |
| Lipid lowering drugs (%) | 4.4 | 5.6 | 3.6 | 5.3 | 4.5 | 3.9 |
| Aspirin (%) | 10.1 | 10.9 | 7.2 | 9.5 | 9.8 | 8.9 |
| Body mass index (kg/m^2^) | 27.2 ± 4.6 | 27.3 ± 5.0 | 27.5 ± 4.7 | 27.5 ± 4.8 | 26.9 ± 4.7 | 27.6 ± 4.8 |
| Waist circumference (cm) | 94.9 ± 11.8 | 94.6 ± 12.7 | 94.4 ± 11.7 | 91.7 ± 13.1 | 89.7 ± 13.3 | 90.0 ± 12.7 |
| SBP (mmHg) | 114 ± 17.8 | 114 ± 17.8 | 114 ± 16.6 | 114 ± 18.1 | 113 ± 17.1 | 114 ± 17.0 |
| DBP (mmHg) | 73.0 ± 10.1 | 72.4 ± 10.9 | 72.2 ± 10.3 | 72.8 ± 10.5 | 72.5 ± 10.7 | 72.3 ± 10.1 |
| FPG (mmol/L) | 94.2 ± 26.0 | 95.8 ± 26.4 | 95.8 ± 24.8 | 94.1 ± 23.9 | 95.7 ± 28.0 | 95.9 ± 28.7 |
| TG to HDL-C ratio | 4.0 ± 3.1 | 4.0 ± 3.2 | 3.8 ± 3.1 | 4.2 ± 3.2 | 3.8 ± 3.0 | 3.8 ± 3.1 |
| Serum creatinine (μmol/L) | 93.2 ± 17.3 | 92.4 ± 16.5 | 90.8 ± 14.4 * | 92.4 ± 16.9 | 92.5 ± 16.0 | 91.5 ± 15.5 |
| eGFR(mL/min/1.73 m^2^) | 77.9 ± 12.2 | 79.5 ± 12.8 | 78.3 ± 12.8 | 78.6 ± 12.4 | 79.1 ± 13.0 | 77.9 ± 12.4 |
| Dietary NO_3_^−^ (mg/day) | 280 ± 61.1 | 432 ± 42.3 | 674 ± 189 | 3.14 ± 104 | 451 ± 117 | 622 ± 218 * |
| Dietary NO_2_^−^ (mg/day) | 6.6 ± 2.1 | 9.4 ± 2.6 | 12.6 ± 3.6 | 7.6 ± 1.5 | 8.7 ± 1.8 | 10.8 ± 3.1 * |
| Incident case of CKD after 5.8 year (%) | 19.6 | 17.3 | 16.7 | 18.5 | 18.0 | 17.0 |

Data are mean ± SD (unless stated otherwise); Analysis of variance or chi-square test were used for continuous and categorical variables, respectively; * *p* < 0.05; SBP: Systolic blood pressure; DBP: Diastolic blood pressure; FPG: Fasting plasma glucose; TG: Triglycerides; eGFR: Estimated glomerular filtration rate; HTN: Hypertension.

**Table S2.** Baseline characteristics of the participants according to follow-up HTN status: Tehran Lipid and Glucose Study 2006–2008 to 2012–2014.

|  | **Non-Incident HTN  (*n* = 1587)** | **Incident HTN  (*n* = 291)** | **Total Population  (*n* = 1878)** |
| --- | --- | --- | --- |
| Age (year) | 35.1 ± 11.9 | 44.3 ± 12.2 * | 36.6 ± 12.4 |
| Men (%) | 41.5 | 50.5 * | 42.9 |
| Smoking (%) | 3.0 | 2.1 | 2.9 |
| Body mass index (kg/m^2^) | 25.8 ± 4.5 | 29.0 ± 4.6 * | 26.3 ± 4.7 |
| Waist circumference (cm) | 85.8 ± 12.8 | 95.2 ± 10.6 * | 87.3 ± 13.0 |
| Systolic blood pressure (mmHg) | 105 ± 11.2 | 115 ± 10.7 * | 107 ± 11.8 |
| Diastolic blood pressure (mmHg) | 69.7 ± 8.6 | 77.0 ± 7.4 * | 70.9 ± 8.8 |
| Fasting plasma glucose (mmol/L) | 4.81 ± 0.70 | 5.18 ± 1.28 * | 4.87 ± 0.83 |
| Triglycerides to HDL-C ratio | 3.16 ± 2.49 | 4.26 ± 2.91 * | 3.3 ± 2.6 |
| Diabetes (%) | 1.8 | 7.4 * | 2.7 |
| Serum creatinine (μmol/L) | 91.4 ± 12.9 | 93.9 ± 16.2 * | 91.7 ± 13.5 |
| eGFR(mL/min/1.73 m^2^) | 81.6 ± 13.2 | 76.5 ± 12.9 * | 80.9 ± 13.3 |
| Dietary NO_3_^−^(mg/day) | 453 ± 187 | 463 ± 194 | 455 ± 188 |
| Dietary NO_2_^−^ (mg/day) | 9.50 ± 3.58 | 9.45 ± 3.78 | 9.47 ± 3.61 |

Data are mean ± SD (unless stated otherwise); Independent *t*-test and chi-square test were used for continuous and categorical variables, respectively; * *p* < 0.05; HTN: Hypertension; eGFR: Estimated glomerular filtration rate.

**Table S3.** Baseline characteristics of the participants according to follow-up CKD status: Tehran Lipid and Glucose Study 2006–2008 to 2012–2014.

|  | **Non-Incident CKD (*n* = 1392)** | **Incident CKD  (*n* = 306)** | **Total Population (*n* = 1780)** |
| --- | --- | --- | --- |
| Age (year) | 33.8 ± 15.3 | 34.3 ± 15.7 | 33.9 ± 15.4 |
| Men (%) | 42.1 | 35.2 * | 40.8 |
| Smoking (%) | 9.5 | 5.8 * | 8.8 |
| Body mass index (kg/m^2^) | 27.1 ± 4.8 | 28.5 ± 4.5 * | 27.4 ± 4.8 |
| Waist circumference (cm) | 89.9 ± 13.3 | 95.0 ± 11.0 * | 90.8 ± 13.1 |
| Systolic blood pressure (mmHg) | 112 ± 16.3 | 122 ± 19.8 * | 114 ± 17.4 |
| Diastolic blood pressure (mmHg) | 72.1 ± 10.3 | 74.7 ± 10.5 * | 72.6 ± 10.5 |
| Fasting blood glucose (mmol/L) | 5.16 ± 1.32 | 5.86 ± 2.05 | 5.29 ± 1.49 |
| Triglycerides to HDL-C ratio | 3.87 ± 3.2 | 4.42 ± 2.86 * | 3.9 ± 3.1 |
| Diabetes (%) | 11.4 | 12.9 | 11.7 |
| Creatinine (μmol/L) | 90.1 ± 13.4 | 91.3 ± 12.7 | 90.2 ± 13.3 |
| eGFR(mL/min/1.73 m^2^) | 80.7 ± 12.5 | 68.9 ± 7.8 * | 78.6 ± 12.7 |
| Dietary NO_3_^−^ (mg/day) | 467 ± 206 | 443 ± 166 * | 462 ± 200 |
| Dietary NO_2_^−^ (mg/day) | 9.64 ± 3.75 | 9.50 ± 3.82 | 9.61 ± 3.76 |

Data are mean ± SD (unless stated otherwise); Independent *t*-test and chi-square test were used for continuous and categorical variables, respectively; * *p* < 0.05; CKD: Chronic kidney disease;
eGFR: Estimated glomerular filtration rate.
